# Ice-Ice Disease Prevalence and Intensity in Eucheumatoid Seaweed Farms: Seasonal Variability and Relationship with the Physicochemical and Meteorological Parameters

**DOI:** 10.3390/plants13152157

**Published:** 2024-08-03

**Authors:** Albaris B. Tahiluddin, Ertugrul Terzi

**Affiliations:** 1 College of Fisheries, Mindanao State University-Tawi-Tawi College of Technology and Oceanography, Sanga-Sanga, Bongao 7500, Philippines; 2 Department of Aquaculture, Institute of Science, Kastamonu University, Kastamonu 37200, Türkiye; 3 Department of Veterinary Medicine, Devrekani TOBB Vocational School, Kastamonu University, Kastamonu 37700, Türkiye; ertugrulterzi@gmail.com

**Keywords:** environmental factors, ice-ice disease occurrence, correlation, *Kappaphycus*, seaweed farming

## Abstract

Since its initial report in the Philippines in 1981, ice-ice disease (IID) remains a substantial threat to the sustainability of eucheumatoid seaweed production. However, comprehensive investigations into the prevalence, intensity, and potential relationships with physicochemical and meteorological parameters are limited, particularly in open-sea farms. This is the first study to conduct a 12-month monitoring of IID prevalence and intensity in eucheumatoid seaweed farms in Tawi-Tawi, Philippines. The research aimed to elucidate seasonal variations and potential associations with physicochemical and meteorological parameters. The findings revealed significant seasonal variations in IID prevalence, with a higher incidence observed during the dry season compared to the wet season. Additionally, deep-water farms exhibited significantly higher prevalence and ice-ice spot numbers per bundle compared to shallow-water farms. Furthermore, *Kappaphycus striatus* displayed a significantly greater ice-ice spot length per bundle compared to *K. alvarezii*. Notably, no interaction effects were observed among season, species, and depth. Interestingly, while no correlations were found between ice-ice disease and most measured physicochemical and meteorological parameters (temperature, salinity, pH, water current velocity, wind speed) or nutrient concentrations (phosphate, nitrate, nitrite), an inverse correlation (*p* < 0.05) emerged between ammonium levels and IID intensity parameters (number and length of ice-ice spot per bundle). Moreover, a positive correlation was observed between monthly rainfall and IID intensity. These findings offer valuable insights into the dynamics of IID in open-sea eucheumatoid seaweed (*Kappaphycus*) farming, highlighting the influence of seasonality, depth, and species susceptibility, as well as the relationship between IID severity and ammonium levels and rainfall.

## 1. Introduction

The cultivation of eucheumatoid seaweeds, encompassing *Kappaphycus* and *Eucheuma*, has seen a recent global increase, tracing its roots to the establishment of the first commercial farm in Tawi-Tawi, Philippines, during the 1970s. This expansion is fueled by the burgeoning global demand for carrageenan [1,2,3]. Carrageenan, an extracted hydrocolloid from eucheumatoid seaweeds, possesses a diverse array of applications across food and non-food industries [4,5,6]. However, intensified farming practices have led to the overcrowding of cultivated seaweed, exacerbating competition for space and nutrients [1,7]. Furthermore, the reliance on vegetative cuttings renders older cultivars highly susceptible to diseases and pests, contributing to frequent disease outbreaks within farms, with ice-ice disease (IID) being particularly noteworthy [8]. 

IID, characterized by depigmentation, whitening, and tissue bleaching that leads to softening and breakdown, is a prevalent disease affecting eucheumatoid seaweed cultivation [9]. It was first documented in the 1980s in Tawi-Tawi, Philippines, the birthplace of commercial eucheumatoid seaweed cultivation. IID has become a global concern due to the expansion of eucheumatoid seaweed farming driven by rising carrageenan demand [7,10]. While the exact cause remains elusive, environmental fluctuations are believed to be crucial contributors. Potential factors include variations in water parameters, such as an elevated temperature, reduced salinity, and low irradiance [11,12], alongside nutrient deficiencies [6,13,14]. Additionally, marine microorganisms, including pathogenic bacteria and fungi, are implicated as co-factors, particularly under environmental stress. Their interactions with abiotic factors can exacerbate disease incidence [9,15,16,17].

In farms, IID weakens and softens the affected thalli, leading to eventual detachment from cultivation lines due to water movement, resulting in significant losses [18]. This, combined with epiphyte presence, poses a substantial threat to eucheumatoid seaweed farming sustainability. Challenges include depleted culture stocks, a declining carrageenan quality, reduced income, and limited job opportunities, particularly impacting marginalized seaweed farmers [9,19]. Notably, IID contributed to a 15% reduction in Philippine *Kappaphycus* production between 2011 and 2013 [20]. Consequently, various mitigation strategies are emerging, such as inorganic nutrient enrichment [1,6,14,17] and biosecurity measures [21,22], demonstrating effectiveness in controlling both IID and epiphyte infestations. Additionally, polyculture with other seaweeds, pre-soaking with antibacterial substances, and proper farm management are suggested as control measures [7].

Despite existing research investigating various environmental stressors potentially linked to IID occurrence [4,9,10,11,12,13,16,18,23,24,25,26], a gap remains in understanding the seasonal variations in IID prevalence and intensity, and their correlations with water parameters. This study aims to address this gap by monitoring the seasonal prevalence and intensity of IID in *Kappaphycus alvarezii* and *K. striatus* farms. Furthermore, it investigates potential correlations with both physicochemical (temperature, salinity, pH, water current velocity) and meteorological factors (wind speed and rainfall).

## 2. Results

**Figure 1:**
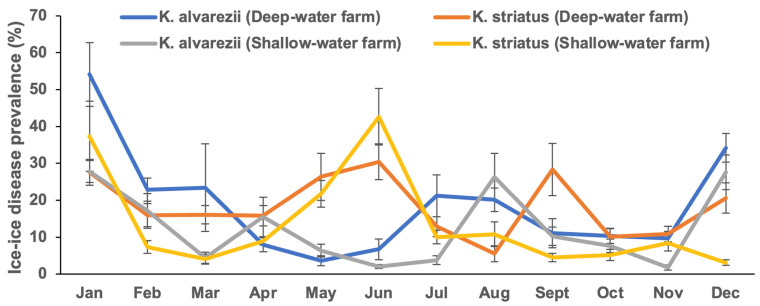
Ice-ice disease prevalence (%) of *Kappaphycus* in deep-water and shallow-water farms (mean ± SE).

Figure 1 (Table S1) shows the seasonal prevalence of IID in *Kappaphycus* farms across different depths. The prevalence ranged from 2.10 ± 0.55% in June (wet season) to 54.11 ± 8.64% in January (dry season). The statistical analysis (Robust ANOVA) revealed significant differences (*p* < 0.05) in the IID prevalence based on depth. Shallow-water farms exhibited a statistically lower prevalence (*p* < 0.05) compared to deep-water farms. Additionally, the prevalence was significantly lower (*p* < 0.05) in the wet season compared to the dry season. Notably, no significant differences (*p* > 0.05) were observed between species, nor were there interaction effects (*p* > 0.05) among depth, season, and species (Table 1).

**Figure 2:**
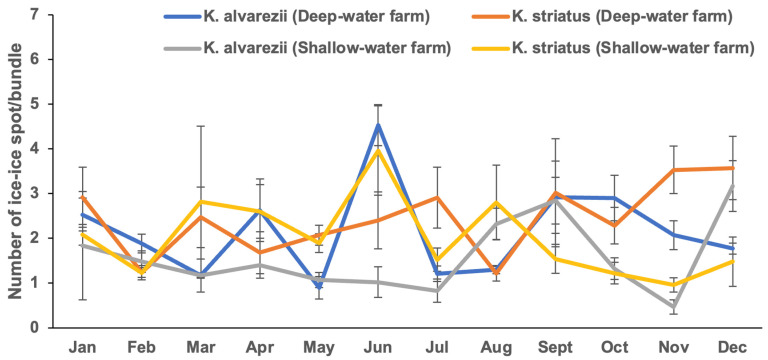
Intensity (number of ice-ice spot per bundle) of ice-ice disease in *Kappaphycus* in deep-water and shallow-water farms (mean ± SE).

**Figure 3:**
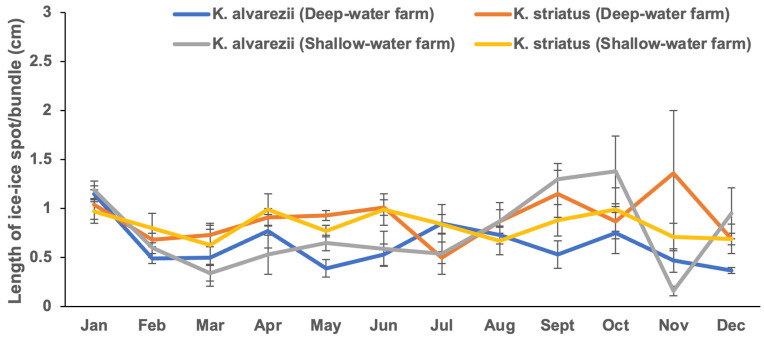
Intensity (length of ice-ice spot per bundle) of ice-ice disease in *Kappaphycus* in deep-water and shallow-water farms (mean ± SE).

Figure 2 (Table S2) and Figure 3 (Table S3) illustrate the intensity levels of IID, expressed as the number and length of ice-ice spots per bundle, across seasons in *Kappaphycus* farms. The number of ice-ice spot per bundle ranged from 0.83 ± 0.26 in July (wet season) to 4.53 ± 0.46 in June (wet season). Shallow-water farms exhibited significantly fewer ice-ice spot per bundle (*p* < 0.05) compared to deep-water farms. However, no significant differences (*p* > 0.05) were found in terms of season or species. Similar to the prevalence, no interaction effects were detected between depth, season, and species (Table 1). Evaluating the intensity based on the length of ice-ice spot per bundle (0.16 ± 0.05–1.38 ± 0.36 cm) revealed a significant difference (*p* < 0.05). *K. striatus* had a significantly higher length of ice-ice spot per bundle compared to *K. alvarezii* (*p* < 0.05). However, neither season nor depth showed significant differences (*p* > 0.05) in the length of ice-ice spot per bundle. Additionally, no interaction effects were observed between these factors (Table 1).

**Figure 4:**
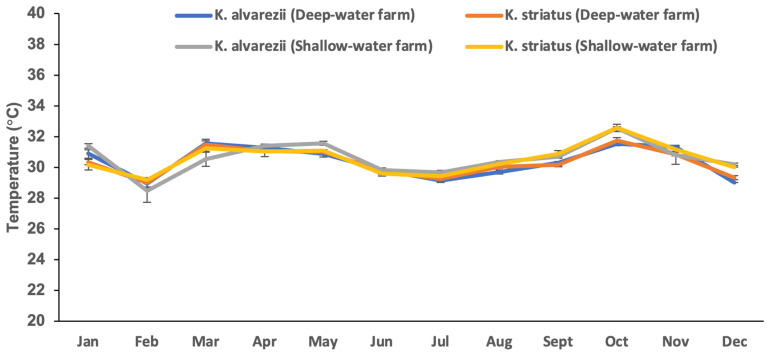
Temperature of the *Kappaphycus* farms during the sampling period (mean ± SE).

**Figure 5:**
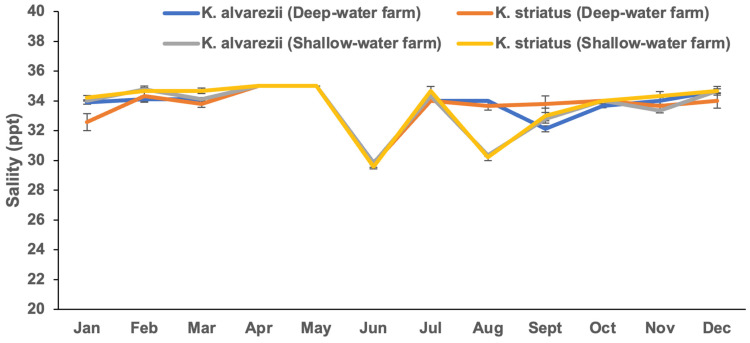
Salinity of the *Kappaphycus* farms during the sampling period (mean ± SE).

**Figure 6:**
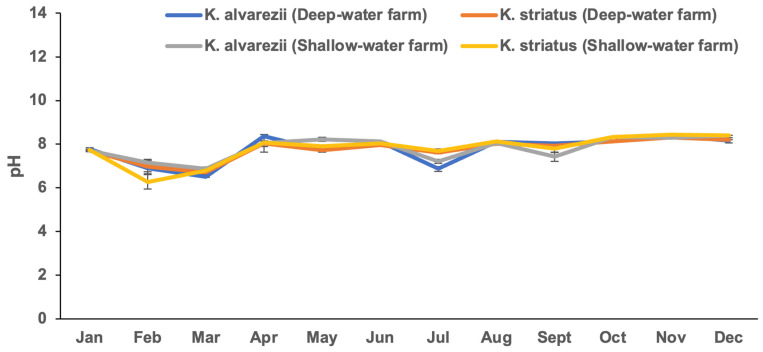
pH levels of the *Kappaphycus* farms during the sampling period (mean ± SE).

**Figure 7:**
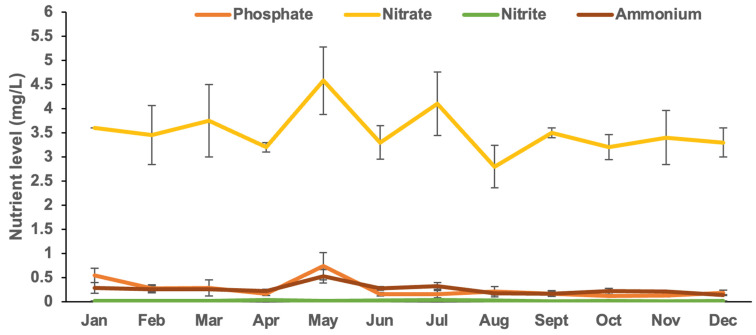
Nutrient levels of *Kappaphycus* farms during the sampling period (mean ± SE).

**Figure 8:**
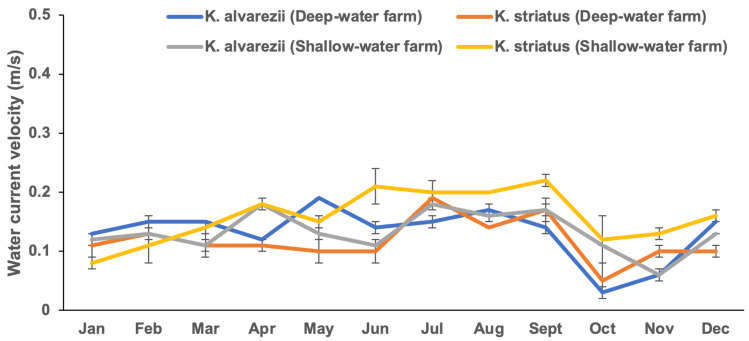
Water current velocity of the *Kappaphycus* farms during the sampling period (mean ± SE).

**Figure 9:**
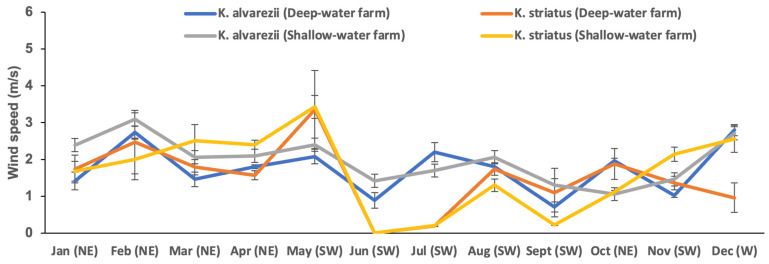
Wind speed and direction of the *Kappaphycus* farms during the sampling period (mean ± SE). NE = Northeast, SW = Southwest, W = West.

**Figure 10:**
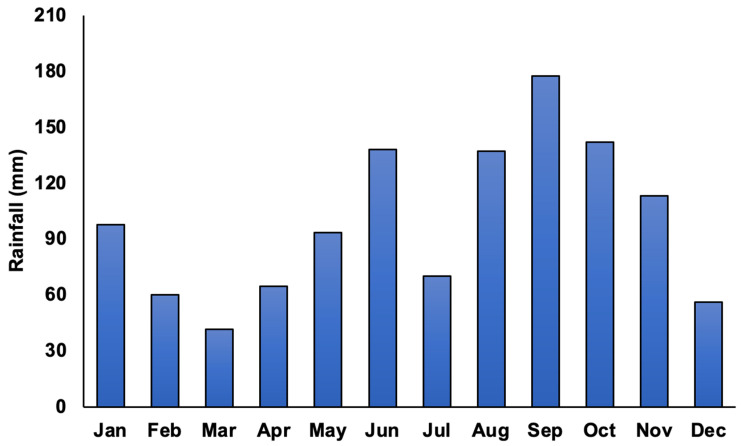
Rainfall (mm) in Sibutu, Tawi-Tawi, Philippines.

The *Kappaphycus* farms’ physicochemical and meteorological data from monthly monitoring are presented in Figure 4, Figure 5, Figure 6, Figure 7, Figure 8, Figure 9 and Figure 10 (Tables S4–S9). The temperature ranged from 28.47 ± 0.76 °C in February to 32.57 ± 0.23 °C in October (Figure 4). The salinity varied between 29.58 ± 0.14 ppt in June and 35.00 ± 0.00 ppt in April (Figure 5). The pH levels fluctuated between 6.27 ± 0.33 in February and 8.43 ± 0.03 in November (Figure 6). Nutrient levels were also monitored, with phosphate fluctuating between 0.12 ± 0.02 and 0.74 ± 0.28 mg L^−1^, nitrate between 2.80 ± 0.44 and 4.58 ± 0.70 mg L^−1^, nitrite between 0.01 ± 0.00 and 0.04 ± 0.00 mg L^−1^, and ammonia between 0.14 ± 0.01 and 0.53 ± 0.14 mg L^−1^ (Figure 7). The water current varied depending on the tides, ranging from 0.06 ± 0.01 m s^−1^ during neap tides to 0.22 ± 0.01 m s^−1^ during spring tides (Figure 8). The wind speeds ranged between 0.89 ± 0.21 and 3.43 ± 0.31 m s^−1^ (Figure 9). As illustrated in Figure 10, rainfall peaked during the wet season.

Table 2 presents the correlation analysis between IID and various physicochemical and meteorological parameters, including the temperature, salinity, pH, nutrient levels (phosphate, nitrate, nitrite, and ammonium), water current velocity, wind speed, and rainfall. It is noteworthy that the prevalence of IID exhibited positive correlations with its own intensity parameters, namely the number and length of ice-ice spots per bundle. However, no significant correlations were observed between IID and the measured physicochemical parameters (temperature, salinity, pH, water current velocity) or wind speed. Furthermore, the analysis revealed no correlations between IID prevalence or intensity and the nutrient concentrations (phosphate, nitrate, nitrite). Interestingly, a negative correlation emerged between the ammonium concentration and both IID intensity parameters (number and length of ice-ice spots per bundle). Additionally, a positive correlation was observed between the length of the ice-ice spot per bundle and the rainfall intensity.

## 3. Materials and Methods

### 3.1. Study Site

**Figure 11:**
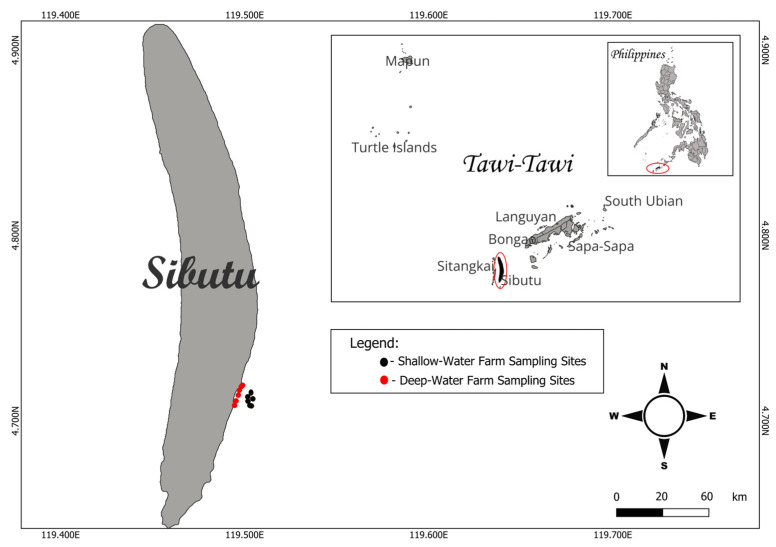
Study sites showing the deep-water farms (red dots) and shallow-water farms (black dots) in Sibutu, Tawi-Tawi, Philippines.

This study was conducted in the municipality of Sibutu, Tawi-Tawi province, Philippines (Figure 11). Sibutu was selected for this study due to its extensive *Kappaphycus* farms and its position as a major supplier of dried *Kappaphycus* in the Philippines. To capture both the dry and wet seasons, a one-year monthly monitoring schedule was implemented from January to December 2023. The dry season encompassed the period from December to May, while the wet or rainy season lasted from June to November.

### 3.2. Farm Description

A total of twelve seaweed farms in Sibutu, Tawi-Tawi, Philippines, were sampled, and Table 3 displays the characteristics of the evaluated farms. Farms were categorized by depth (shallow and deep-water farms) and seaweed species (*K. alvarezii* and *K. striatus*). The evaluations were conducted in triplicate for each farm, with three to five random lines assessed for each species within each farm. All the surveyed *Kappaphycus* farms were enriched with inorganic fertilizer using ammonium phosphate (16-20-0, N-P-K) by the farmers, and the detailed procedure was previously reported [14]. The shallow-water farms, characterized by a depth ranging from 38 to 107 cm, utilized the modified fixed-off bottom method. In contrast, the deep-water farms, with a depth ranging from 177 to 359 cm, employed the floating method. The modified fixed-off bottom method involved stakes installed on both sides of the seabed with floaters (primarily empty plastic bottles). On the other hand, the floating method utilized floaters supported by anchors (rocks or sandbags) at each end [4,26]. 

### 3.3. Ice-Ice Disease Monitoring

IID presence was monitored in different farms through visual inspection monthly over a one-year period. Indicators such as the presence of white and softened thalli were used to identify infected seaweed bundles [14]. The number of diseased bundles was recorded per line, with individual lines containing 29 to 99 bundles. The IID prevalence (%) was calculated as the proportion of IID-infected seaweed bundles within a line to the total number of bundles within that same line. This proportion was then multiplied by 100 to express the prevalence as a percentage, as indicated in the formula below [8,26].

$$\text{Ice-ice}\;\mathrm{disease}\;\mathrm{prevalence}\;\left(\%\right)=\frac{\mathrm{Number}\;\mathrm{of}\;\mathrm{infected}\;\mathrm{seaweed}\;\mathrm{bundles}\;\mathrm{within}\;a\;\mathrm{line}}{\mathrm{Total}\;\mathrm{number}\;\mathrm{of}\;\mathrm{seaweed}\;\mathrm{bundles}\;\mathrm{within}\;a\;\mathrm{line}}\times 100$$

The intensity of IID was expressed by recording both the number and the length (cm) of ice-ice spots per infected bundle using a ruler. Infected bundles containing ice-ice spots were carefully counted, and the length of each spot was measured (cm) using a ruler. The average number and length of ice-ice spots per bundle were calculated for each farm [14]. 

### 3.4. Physicochemical Parameter Assessment

Physicochemical parameters, including the temperature, salinity, and pH, were monitored monthly throughout the year of IID monitoring. Measurements (n = 3) were taken once a month. The temperature and pH were measured in situ using a thermometer and pH meter (Polsinelli, Kansas, MO, USA), respectively, submerged at a depth of 7–12 cm adjacent to the cultivated lines. The salinity was measured using a refractometer (Atago Master, Tokyo, Japan) from water samples collected at the same location. The water depth and current were determined using a calibrated rope and a fabricated drogue, respectively.

Meteorological parameters, including the wind speed and direction, and rainfall, were monitored throughout the year. The wind speed and direction were measured using a digital anemometer (BENETECH, Shenzhen, China), while rainfall was collected daily using a rain gauge. The monthly phosphate, nitrate, nitrite, and ammonia concentrations in the farm waters (n = 3, e.g., deep, mid, and shallow-waters) were determined by the PhosVer 3 Method, Cadmium Reduction Method, Diazotization method, and Salicylate Method, respectively, using Hach Lange tests and a UV-VIS DR6000 spectrophotometer (Hach Lange, Loveland, CO, USA).

### 3.5. Statistical Analysis 

Data (n = 3) were expressed as mean ± SE. Initially, the normality and homogeneity of the data were assessed using the Shapiro–Wilk test and Levene’s test, respectively, in SPSS statistical software (SPSS version 20, SPSS Inc., Chicago, IL, USA). As the data were found to be non-normally distributed and exhibit unequal variances, the statistical analyses were conducted using the Robust ANOVA (Walrus) implemented in Jamovi (version 2.3.28.0, The Jamovi Project, Sydney, Australia). Robust ANOVA (Walrus), a reliable alternative to classic ANOVA, analyzes group differences even with non-normal data or unequal variances. Pearson correlation coefficients were calculated in SPSS statistical software (SPSS version 20, SPSS Inc., Chicago, IL, USA) to assess the correlations between IID parameters (prevalence, number of ice-ice spot per bundle, and length of ice-ice spot per bundle) and both physicochemical and meteorological parameters.

## 4. Discussion

The prevalence and intensity (number and length of ice-ice spots per bundle) of IID were monitored on a monthly basis throughout the year to capture seasonal variations and their relationships with physicochemical and meteorological parameters. All surveyed farms, regardless of species or depth, encountered IID throughout the year. This finding aligns with previous reports documenting the widespread occurrence of IID in the Philippines [6,18,25,27,28,29,30,31] and globally [7,22,23,32,33,34]. Notably, a positive correlation was observed between the prevalence and intensity of IID, indicating that a higher prevalence coincides with greater intensity. This suggests that as the disease progresses, it intensifies, potentially leading to complete farm loss due to currents or the harvesting of infected seaweed. Faisan et al. [18] proposed that the progressive spread of IID to other thalli regions and increased susceptibility to breakage by water currents contribute to this intensification.

Significant seasonal variations in IID prevalence, with a higher prevalence observed during the dry season (December to May) compared to the wet season (June to November), were observed in the present study. Largo et al. [35] reported a similar trend, observing a remarkable 99% prevalence of IID in a eucheumatoid seaweed farm in Zanzibar, Tanzania, during the dry season (February to March). Additionally, farmers in the southern Philippines associate the “bad season” with the hot and dry months (December to May) [3].

While no correlations were found between IID and the concentrations of phosphate, nitrate, and nitrite, an inverse correlation emerged between the intensity of IID (number and length of ice-ice spots per bundle) and the ammonium concentration. This suggests that a decreasing ammonium concentration in the farm coincides with an increasing IID intensity, indicating the potential role of ammonium as a crucial nutrient influencing disease severity in *Kappaphycus* farms. This finding aligns with previous reports demonstrating that inorganic nutrient enrichment using ammonium phosphate significantly reduces IID prevalence [3,6,14]. The effectiveness of this strategy is attributed to its ability to elevate the internal nitrogen content, which is critical for seaweed growth [36]. It is noteworthy that, compared to other nitrogen sources, ammonium is readily assimilated by most seaweeds due to its preferred form [37,38].

This study revealed a significant difference in IID prevalence and intensity (number of ice-ice spot per bundle) between deep and shallow-water farms. Deep-water farms exhibited a higher prevalence and intensity compared to shallow-water farms. While previous reports documented no significant differences in prevalence [18,26], they relied on either one-time monitoring [18] or a limited three-month period [26]. This one-year study provides evidence for the potential impact of depth on IID dynamics.

The lack of correlation between IID and the measured salinity, despite one-time monthly surveys, suggests that further investigations considering more frequent salinity measurements are needed. However, the proximity of deep-water farms to stilt houses and the landmass raises concerns about potential salinity fluctuations. During the rainy season, run-offs reaching deeper areas could potentially lower the salinity compared to shallow farms located further away. Additionally, freshwater discharges from stilt houses might contribute to salinity variations in deep-water farms. Furthermore, the floating cultivation method employed in deep-water farms, regardless of tides, keeps the seaweeds near the surface, increasing their vulnerability to fluctuations in water parameters like salinity and temperature. This contrasts with shallow reef areas where the modified fixed-off bottom method allows submergence during high tides and maintains a greater distance from landmass. The aforementioned environmental factors associated with deep-water farm placement, coupled with the continuous surface exposure of seaweeds due to the floating cultivation method, present a compelling explanation for the observed higher prevalence of IID in these settings compared to shallow-water farms. This hypothesis is further supported by research from Faisan et al. [18], who suggest that environmental stressors, including prolonged deviations from normal rainfall patterns, elevated near-surface water temperatures, and diminished wind speeds, can contribute to seaweed stress, potentially leading to an increased incidence of IID.

While no significant difference was observed in IID prevalence or the number of ice-ice spot per bundle between *K. striatus* and *K. alvarezii*, our results revealed a significantly higher length of ice-ice spot per bundle in *K. striatus* compared to *K. alvarezii*. This suggests that *K. striatus*, when infected, develops wider IID lesions compared to *K. alvarezii*, aligning with observations from local farmers. This finding contradicts the findings of a study by Hurtado et al. [39], where the *K. striatus* sacol variety was reported to be more resistant than the *K. alvarezii* var. *tambalang*. However, their report relied on anecdotal farmer observations rather than scientific data. Further investigations are crucial to understand the distinct response of *Kappaphycus* species to IID infection intensity. Speculatively, the observed difference might be attributed to species morphology. *K. alvarezii* has a slender and smooth structure, while *K. striatus* forms bushier branches near the tips and is more compact, especially in the apical region. This morphology in *K. striatus* may allow easier and faster pathogen spread between closely packed branches compared to the smooth and slender *K. alvarezii*. Nevertheless, this hypothesis requires further research.

Except for rainfall and ammonium levels, the one-time monitoring of physicochemical parameters (temperature, salinity, pH, water current velocity) revealed no correlations with IID prevalence or intensity (number and length of ice-ice spots per bundle). Although correlations were not observed in the present study, previous controlled laboratory experiments suggest that changes in water parameters, such as the increased sea surface temperature (33–35 °C), may contribute to IID development [7,11]. Additionally, it was proved that biological factors like marine bacteria belonging to the *Vibrio* and *Cytophaga* groups play a crucial role in IID development and act synergistically with stressful environmental conditions to exacerbate the disease [7,15]. Largo et al. [11] demonstrated that salinity reduction below 20 ppt can induce the whitening of eucheumatoid seaweed thalli, potentially contributing to IID development. Interestingly, our study revealed a positive correlation between the length of ice-ice spot per bundle (IID intensity) and rainfall, supporting the findings of Largo et al. [11], who stated that the salinity reduction (like those caused by heavy rainfall) resulted in IID; this aligns with local farmer observations.

## 5. Conclusions

In open-sea *Kappaphycus* farms, ice-ice disease (IID) is prevalent throughout the year, with a peak occurring during the dry season (December to May). The depth of the farm was identified as a significant factor, with deep-water farms exhibiting higher IID susceptibility compared to shallow-water farms. The study revealed a difference in the intensity of IID between the two *Kappaphycus* species, with *K. alvarezii* showing a higher degree of resistance than *K. striatus*, as evidenced by the measured length of ice-ice spot per bundle. While no correlations were observed between IID prevalence and intensity and the measured physicochemical parameters (temperature, salinity, pH, etc.) or nutrient concentrations (phosphate, nitrate, and nitrite) across the various farms investigated, an inverse correlation was found between the intensity of IID (number and length of ice-ice spots per bundle) and ammonium levels. There was a positive correlation between rainfall and the intensity of IID, as measured by the length of ice-ice spot per bundle. 

This study provides valuable insights into the seasonal patterns, depth-related variations, and species-specific impacts on IID in open-sea *Kappaphycus* farms. However, the absence of data on farm management practices, such as seaweed density per line, cleaning procedures, etc., represents a limitation, as these practices might confound the observed relationships with environmental factors. Future research should incorporate these variables to develop a more comprehensive understanding of IID dynamics and to inform effective management strategies for sustainable seaweed cultivation.

Based on the current findings, to minimize IID occurrence in cultivated *Kappaphycus* farms, farmers in the study area could implement several strategies. Firstly, reducing exposure to the sea surface by minimizing floaters or submerging cultivation lines deeper could mitigate salinity fluctuations during the rainy season, a known IID trigger. Secondly, cultivating less susceptible species, such as *K. alvarezii* instead of *K. striatus*, may reduce IID prevalence. Additionally, as nutrient (ammonium) deficiency is linked to IID, nutrient enrichment could be explored as a potential mitigation measure. Lastly, strict adherence to biosecurity measures is essential, given their demonstrated efficacy in reducing IID incidence.

## Figures and Tables

**Table 1:** Statistical analysis (*p* values) of the obtained data on the prevalence and its intensity levels using the Robust ANOVA (Walrus). ** = significant at *p* < 0.01; * = significant at *p* < 0.05.

Factors	Ice-Ice Disease Prevalence (%)	No. of Ice-Ice Spot/Bundle	Length of Ice-Ice Spot/Bundle
Depth	*p* (<0.001) **	*p* (0.045) *	*p* (0.770)
Season	*p* (0.015) *	*p* (0.840)	*p* (0.360)
Species	*p* (0.993)	*p* (0.154)	*p* (0.008) **
Depth × Season	*p* (0.365)	*p* (0.515)	*p* (0.976)
Depth × Species	*p* (0.746)	*p* (0.423)	*p* (0.311)
Season × Species	*p* (0.172)	*p* (0.847)	*p* (0.996)
Depth × Season × Species	*p* (0.620)	*p* (0.937)	*p* (0.684)

**Table 2:** Pearson correlation coefficients (r) and *p* values. ** = significant at *p* < 0.01; * = significant at *p* < 0.05.

	Ice-Ice Disease Prevalence (%)	No. of Ice-Ice Spot/Bundle	Length of Ice-Ice Spot/Bundle	Temperature	Salinity	pH	Current Velocity	Wind Speed	Rainfall	Phosphate	Nitrate	Nitrite	Ammonium
Ice-ice disease prevalence (%)	1	0.560 ** (0.000)	0.217 ** (0.009)	−0.157 (0.060)	−0.042 (0.617)	0.000 (0.999)	−0.052 (0.536)	0.022 (0.791)	0.002 (0.982)	0.095 (0.589)	−0.050 (0.783)	−0.198 (0.270)	−0.254 (0.160)
No. of ice-ice spot/bundle	0.560 (0.000)	1	0.105 (0.211)	−0.012 (0.885)	−0.109 (0.195)	0.111 (0.186)	0.115 (0.168)	−0.132 (0.115)	0.133 (0.111)	−0.240 (0.165)	−0.235 (0.187)	−0.303 (0.086)	−0.422 * (0.016)
Length of ice-ice spot/bundle	0.217 (0.009)	0.105 (0.211)	1	0.158 (0.059)	−0.126 (0.133)	0.097 (0.248)	−0.099 (0.237)	−0.071 (0.396)	0.232 ** (0.005)	−0.117 (0.503)	−0.227 (0.204)	−0.196 (0.273)	−0.359 * (0.047)
Temperature	−0.157 (0.060)	−0.012 (0.885)	0.158 (0.059)	1	0.007 (0.935)	0.248 ** (0.003)	−0.102 (0.225)	−0.145 (0.082)	0.182 * (0.028)	0.092 (0.601)	−0.018 (0.919)	−0.075 (0.678)	−0.035 (0.849)
Salinity	−0.042 (0.617)	−0.109 (0.195)	−0.126 (0.133)	0.007 (0.935)	1	−0.011 (0.893)	0.099 (0.240)	0.484 ** (0.000)	−0.500 ** (0.000)	0.331 (0.069)	0.137 (0.446)	0.281 (0.114)	0.327 (0.068)
pH	0.000 (0.999)	0.111 (0.186)	0.097 (0.248)	0.248 ** (0.003)	−0.011 (0.893)	1	0.068 (0.415)	−0.169 * (0.042)	0.437 ** (0.000)	−0.044 (0.802)	−0.311 (0.078)	−0.041 (0.833)	−0.263 (0.146)
Current velocity	−0.052 (0.536)	0.115 (0.168)	−0.099 (0.237)	−0.102 (0.225)	0.099 (0.240)	0.068 (0.415)	1	−0.106 (0.204)	−0.076 (0.367)	−0.037 (0.833)	0.236 (0.236)	0.003 (0.985)	−0.117 (0.523)
Wind speed	0.022 (0.791)	−0.132 (0.115)	−0.071 (0.396)	−0.145 (0.082)	0.484 ** (0.000)	−0.169 * (0.042)	−0.106 (0.204)	1	−0.369 ** (0.000)	0.214 (0.218)	0.057 (0.754)	0.069 (0.703)	0.346 (0.053)
Rainfall	0.002 (0.982)	0.133 (0.111)	0.232 ** (0.005)	0.182 * (0.028)	−0.500 ** (0.000)	0.437 ** (0.000)	−0.076 (0.367)	−0.369 ** (0.000)	1	−0.135 (0.440)	−0.168 (0.435)	−0.144 (0.425)	−0.202 (0.267)
Phosphate	0.095 (0.589)	−0.240 (0.165)	−0.117 (0.503)	0.092 (0.601)	0.331 (0.069)	−0.044 (0.802)	−0.037 (0.833)	0.214 (0.218)	−0.135 (0.440)	1	0.406 * (0.019)	−0.143 (0.429)	0.433 ** (0.015)
Nitrate	−0.050 (0.783)	−0.235 (0.187)	−0.227 (0.204)	−0.018 (0.919)	0.137 (0.446)	−0.311 (0.078)	0.236 (0.236)	0.057 (0.754)	−0.168 (0.349)	0.406 * (0.019)	1	0.218 (0.224)	0.669 ** (0.000)
Nitrite	−0.198 (0.270)	−0.303 (0.086)	−0.196 (0.273)	−0.075 (0.678)	−0.041 (0.833)	−0.041 (0.833)	0.003 (0.985)	0.069 (0.703)	−0.144 (0.425)	−0.143 (0.429)	0.218 (0.224)	1	0.186 (0.334)
Ammonium	−0.254 (0.160)	−0.422 * (0.016)	−0.359 * (0.047)	−0.035 (0.849)	−0.263 (0.146)	−0.263 (0.146)	−0.117 (0.523)	0.346 (0.053)	−0.202 (0.425)	0.433 * (0.015)	0.669 ** (0.000)	0.186 (0.334)	1

**Table 3:** Characteristics of the monitored *Kappaphycus* farms.

Farm	Characteristics					
	Depth (cm)	Farming Method	No. of Surveyed Farms	No. of Surveyed Lines per Farm	No. of Bundles per Line	Inorganic Nutrient Enrichment
Deep-water						
*K. alvarezii*	244–359	Floating	3	3–5	29–93	Yes
*K. striatus*	177–303	Floating	3	3–5	30–89	Yes
Shallow-water						
*K. alvarezii*	38–122	Modified fixed-off bottom	3	3–5	23–99	Yes
*K. striatus*	50–107	Modified fixed-off bottom	3	3–5	30–84	Yes

## Data Availability

Data are contained in the article.

## Supplementary Materials

The following supporting information can be downloaded at: https://www.mdpi.com/article/10.3390/plants13152157/s1, Table S1: Data on the prevalence of ice-ice disease; Table S2: Data on the number of ice-ice spot per bundle; Table S3: Data on the length of ice-ice spot per bundle; Table S4: Data on the temperature of *Kappaphycus* farms; Table S5: Data on the salinity of *Kappaphycus* farms; Table S6: Data on pH levels of *Kappaphycus* farms; Table S7: Data on nutrient levels of *Kappaphycus* farms; Table S8: Data on water current velocity of *Kappaphycus* farms; Table S9: Data on wind speed and direction of *Kappaphycus* farms.
