# Supplementary material for: Ice-Ice Disease Prevalence and Intensity in Eucheumatoid Seaweed Farms: Seasonal Variability and Relationship with the Physicochemical and Meteorological Parameters
Source: Plants (Basel). 2024 Aug 3;13(15):2157. doi: 10.3390/plants13152157 (PMC11314110; doi:10.3390/plants13152157)
Supplement: Supplementary file 1 [file plants-13-02157-s001.zip › plants-3103118-supplementary.pdf]

Supplementary Materials

Table S1. Prevalence of ice-ice disease of *Kappaphycus* in deep and shallow farms (mean ± SE).

**Farm**	**Ice-ice disease prevalence (%)**											
	Jan	*Feb*	*Mar*	*Apr*	*May*	*Jun*	*Jul*	*Aug*	*Sep*	*Oct*	*Nov*	*Dec*
**Deep**												
K. alvarezii	54.11 ± 8.64	22.92 ± 3.10	23.45±11.89	7.96±1.88	3.62±1.37	6.75±2.83	21.24±5.67	20.15±3.17	11.08±3.97	10.34±1.98	9.76±1.84	34.24±3.85
K. striatus	27.62 ± 3.50	15.98 ± 3.15	16.10 ± 2.49	15.88±2.74	26.40±6.37	30.46±4.84	12.91±2.70	5.55±2.13	28.35±7.08	10.11±2.33	10.87±2.07	20.54±4.14
**Shallow**												
K. alvarezii	27.80 ± 2.96	17.13 ± 4.71	4.47 ± 1.51	15.52±5.30	6.41±1.74	2.10±0.55	3.78±1.20	26.34±6.41	10.30±2.48	7.69±1.80	1.79±0.72	27.64±4.68
K. striatus	37.39 ± 9.51	7.37 ± 1.75	4.06 ± 1.36	8.99±1.06	21.77±3.62	42.64±7.68	10.04±1.85	10.80±3.37	4.51±1.14	5.20±1.53	8.40±2.14	3.13±0.74

Table S2. Intensity (number of ice-ice spot per bundle) of ice-ice disease of *Kappaphycus* in deep and shallow farms (mean ± SE).

**Farm**	**Number of ice-ice spot/bundle**											
	Jan	*Feb*	*Mar*	*Apr*	*May*	*Jun*	*Jul*	*Aug*	*Sep*	*Oct*	*Nov*	*Dec*
**Deep**												
K. alvarezii	2.53 ± 0.37	1.88 ± 0.21	1.17±0.65	2.63±0.70	0.90±0.25	4.53±0.46	1.21±0.17	1.30±0.09	2.96±0.81	2.90±0.51	2.07±0.32	1.77±0.12
K. striatus	2.92 ± 0.67	1.24 ± 0.11	2.47±0.68	1.68±0.47	2.07±0.22	2.40±0.64	2.91±0.68	1.21±0.16	3.02±1.21	2.28±0.41	3.53±0.53	3.57±0.71
**Shallow**												
K. alvarezii	1.84 ± 0.21	1.48 ± 0.24	1.17±0.37	1.40±0.29	1.07±0.17	1.02±0.34	0.83±0.26	2.32±0.35	2.84±0.52	1.32±0.24	0.47±0.16	3.17±0.57
K. striatus	2.08 ± 0.23	1.23 ± 0.16	2.82±1.69	2.60±0.60	1.89±0.21	3.96±1.00	1.52±0.26	2.80±0.84	1.54±0.32	1.22±0.29	0.96±0.16	1.48±0.55

Table S3. Intensity (length of ice-ice spot per bundle) of ice-ice disease of *Kappaphycus* in deep and shallow farms (mean ± SE).

**Farm**	**Length of ice-ice spot/bundle (cm)**											
	Jan	*Feb*	*Mar*	*Apr*	*May*	*Jun*	*Jul*	*Aug*	*Sep*	*Oct*	*Nov*	*Dec*
**Deep**												
K. alvarezii	1.15 ± 0.08	0.49 ± 0.05	0.5±0.29	0.77±0.17	0.39±0.09	0.53±0.11	0.85±0.19	0.73±0.09	0.53±0.14	0.75±0.21	0.47±0.12	0.37±0.03
K. striatus	1.04 ± 0.15	0.68 ± 0.07	0.73±0.12	0.91±0.09	0.93±0.05	1.01±0.08	0.50±0.06	0.87±0.12	1.15±0.24	0.87±0.18	1.36±0.64	0.69±0.06
**Shallow**												
K. alvarezii	1.19± 0.09	0.60 ± 0.10	0.34±0.08	0.53±0.20	0.65±0.08	0.59±0.18	0.54±0.21	0.87±0.19	1.30±0.16	1.38±0.36	0.16±0.05	0.95±0.26
K. striatus	0.97 ± 0.12	0.80 ± 0.15	0.63±0.20	0.99±0.16	0.77±0.06	0.99±0.16	0.84±0.10	0.67±0.14	0.88±0.16	0.99±0.22	0.71±0.14	0.69±0.15

Table S4. Temperature of the *Kappaphycus* farms during the sampling period (mean ± SE).

**Farm**	**Temperature (°C)**											
	Jan	*Feb*	*Mar*	*Apr*	*May*	*Jun*	*Jul*	*Aug*	*Sep*	*Oct*	*Nov*	*Dec*
**Deep**												
K. alvarezii	30.89 ± 0.26	28.94 ± 0.18	31.56 ± 0.28	31.28 ± 0.11	30.87±0.05	29.83±0.07	29.14±0.06	29.68±0.14	30.29±0.09	31.53±0.06	31.31±0.13	29.00±0.00
K. striatus	30.33 ± 0.17	29.00 ± 0.29	31.47 ± 0.26	31.06 ± 0.10	31.00±0.00	29.75±0.20	29.25±0.14	30.04±0.20	30.17±0.12	31.73±0.20	30.86±0.12	29.33±0.14
**Shallow**												
K. alvarezii	31.39 ± 0.14	28.47 ± 0.76	30.53 ± 0.46	31.39 ± 0.13	31.56±0.13	29.81±0.05	29.67±0.13	30.34±0.07	30.69±0.11	32.54±0.10	30.79±0.60	30.19±0.08
K. striatus	30.17 ± 0.33	29.17 ± 0.17	31.25 ± 0.22	31.03 ± 0.34	31.08±0.07	29.58±0.14	29.42±0.22	30.22±0.22	30.88±0.21	32.57±0.23	31.17±0.14	30.00±0.00

Table S5. Salinity of the *Kappaphycus* farms during the sampling period (mean ± SE).

Farm	Salinity (ppt)											
	Jan	*Feb*	*Mar*	*Apr*	*May*	*Jun*	*Jul*	*Aug*	*Sep*	*Oct*	*Nov*	*Dec*
**Deep**												
K. alvarezii	33.89 ± 0.11	34.11 ± 0.20	34.11 ± 0.00	35.00 ± 0.00	35.00±0.00	29.83±0.02	34.00±0.00	34.00±0.00	32.11±0.20	33.67±0.13	34.00±0.22	34.67±0.13
K. striatus	32.57 ± 0.58	34.33 ± 0.33	33.78±0.22	35.00 ± 0.00	35.00±0.00	29.75±0.20	34.00±0.00	33.67±0.29	33.78±0.56	34.00±0.00	33.67±0.29	34.00±0.50
**Shallow**												
K. alvarezii	34.00 ± 0.00	34.78 ± 0.11	34.11±0.11	35.00 ± 0.00	35.00±0.00	29.81±0.05	34.33±0.13	30.34±0.07	32.78±0.11	34.00±0.00	33.33±0.13	34.67±0.13
K. striatus	34.22 ± 0.15	34.67 ± 0.33	34.67±0.19	35.00 ± 0.00	35.00±0.00	29.58±0.14	34.67±0.29	30.22±0.22	33.00±0.50	34.00±0.00	34.33±0.29	34.67±0.29

Table S6. pH levels of the *Kappaphycus* farms during the sampling period (mean ± SE).

Farm	pH											
	Jan	*Feb*	*Mar*	*Apr*	*May*	*Jun*	*Jul*	*Aug*	*Sep*	*Oct*	*Nov*	*Dec*
**Deep**												
K. alvarezii	7.77 ± 0.06	6.90 ± 0.17	6.51±0.03	8.36 ± 0.08	7.72±0.02	8.10±0.00	6.87±0.11	8.10±0.02	8.04±0.04	8.13±0.01	8.37±0.05	8.17±0.12
K. striatus	7.71 ± 0.05	6.97 ± 0.33	6.70±0.10	8.04 ± 0.41	7.73±0.10	7.97±0.03	7.61±0.05	8.01±0.05	7.92±0.06	8.13±0.03	8.30±0.00	8.20±0.00
**Shallow**												
K. alvarezii	7.71 ± 0.06	7.16 ± 0.09	6.88±0.04	8.06 ± 0.09	8.22±0.09	8.13±0.01	7.21±0.09	8.06±0.01	7.43±0.21	8.33±0.02	8.30±0.00	8.40±0.00
K. striatus	7.75 ± 0.02	6.27 ± 0.33	6.78±0.17	8.08 ± 0.19	7.90±0.06	8.03±0.02	7.69±0.09	8.12±0.03	7.80±0.18	8.33±0.03	8.43±0.03	8.40±0.00

Table S7. Nutrient levels of *Kappaphycus* farms (mean ± SE).

Farm	Nutrient level (mg L^{-1})											
	Jan	*Feb*	*Mar*	*Apr*	*May*	*Jun*	*Jul*	*Aug*	*Sep*	*Oct*	*Nov*	*Dec*
Phosphate PO^{4}-P	0.55 ± 0.15	0.28 ± 0.07	0.29± 0.17	0.17± 0.04	0.74 ± 0.28	0.16 ± 0.04	0.16 ± 0.07	0.21 ± 0.11	0.17 ± 0.06	0.12 ± 0.02	0.13 ± 0.03	0.19 ± 0.05
Nitrate (NO_3-N)	3.60 ± 0.00	3.45 ± 0.61	3.75± 0.75	3.20± 0.10	4.58 ± 0.70	3.30 ± 0.35	4.10 ± 0.66	2.80 ± 0.44	3.50 ± 0.10	3.20 ± 0.26	3.40 ± 0.56	3.30 ± 0.30
Nitrite (NO_2-N)	0.02 ± 0.00	0.02 ± 0.00	0.02± 0.00	0.04± 0.01	0.02 ± 0.01	0.03 ± 0.01	0.04 ± 0.02	0.03 ± 0.01	0.01 ± 0.00	0.02 ± 0.00	0.01 ± 0.00	0.02 ± 0.00
Ammonium NH_4-N	0.29 ± 0.11	0.26 ± 0.07	0.26± 0.02	0.22± 0.04	0.53 ± 0.14	0.28 ± 0.04	0.33 ± 0.07	0.18 ± 0.04	0.17 ± 0.04	0.22 ± 0.06	0.21 ± 0.00	0.14 ± 0.01

Table S8. Water current velocity of the *Kappaphycus* farms during the sampling period (mean ± SE).

Farm	Water current velocity (m s^{-1})											
	Jan	*Feb*	*Mar*	*Apr*	*May*	*Jun*	*Jul*	*Aug*	*Sep*	*Oct*	*Nov*	*Dec*
**Deep**												
K. alvarezii	0.13 ± 0.00	0.15 ± 0.01	0.15± 0.00	0.12 ±0.00	0.19± 0.00	0.14± 0.01	0.15± 0.01	0.17±0.01	0.14±0.01	0.03±0.01	0.06±0.01	0.15±0.00
K. striatus	0.11 ± 0.02	0.13 ± 0.02	0.11± 0.02	0.11 ±0.01	0.10± 0.02	0.10± 0.02	0.19± 0.01	0.14±0.00	0.17±0.02	0.05±0.03	0.10±0.43	0.01±0.01
**Shallow**												
K. alvarezii	0.12 ± 0.01	0.13 ± 0.01	0.11± 0.01	0.18 ±0.00	0.13± 0.01	0.11± 0.00	0.18± 0.01	0.16±0.01	0.17±0.01	0.11±0.00	0.06±0.01	0.14±0.00
K. striatus	0.08 ± 0.01	0.11 ± 0.03	0.14± 0.01	0.18 ±0.01	0.15± 0.01	0.21± 0.03	0.20±0.02	0.20±0.00	0.22±0.01	0.12±0.04	0.13±0.01	0.16±0.01

Table S9. Wind speed and direction of the *Kappaphycus* farms during the sampling period (mean ± SE).

Farm	Wind speed (m s^{-1}) and direction											
	Jan	*Feb*	*Mar*	*Apr*	*May*	*Jun*	*Jul*	*Aug*	*Sep*	*Oct*	*Nov*	*Dec*
	Northeast	*Northeast*	*Northeast*	*Northeast*	*Southwest*	*Southwest*	*Southwest*	*Southwest*	*Southwest*	*Northeast*	*Southwest*	*West*
**Deep**												
K. alvarezii	1.42 ± 0.24	2.74 ± 0.16	1.46±0.20	1.80 ± 0.05	2.08±0.20	0.89±0.21	2.20±0.26	1.80±0.08	0.71±0.14	1.96±0.07	1.02±0.05	2.80±0.15
K. striatus	1.74 ± 0.38	2.47 ± 0.86	1.79±0.21	1.57 ± 0.12	3.36±1.06	0.00±0.00	0.19±0.01	1.74±0.17	1.10±0.66	1.88±0.42	1.36±0.18	0.96±0.40
**Shallow**												
K. alvarezii	2.39 ± 0.23	3.09 ± 0.22	2.06±0.54	2.10 ± 0.11	2.40±0.20	1.42±0.14	1.70±0.07	2.06±0.11	1.30±0.21	1.06±0.08	1.46±0.08	2.71±0.14
K. striatus	1.68 ± 0.27	2.00 ± 0.55	2.51±0.44	2.40 ± 0.12	3.43±0.31	0.00±0.00	0.20±0.02	1.30±0.17	0.22±0.01	1.12±0.04	2.14±0.19	2.56±0.37
